# Individually Recognized Functional Ingredients of Korean Health Functional Foods: Functional Classification, Regulatory Context, and Clinical Implication

**DOI:** 10.3390/nu18040637

**Published:** 2026-02-15

**Authors:** Pureum Kang, So Yeon Kim, Jiayu Shi, Young Hee Choi, Young-Won Chin

**Affiliations:** 1 College of Pharmacy and Integrated Research Institute for Drug Development, Dongguk University_Seoul, 32 Dongguk-lo, Ilsandong-gu, Goyang-si 10326, Gyeonggi-do, Republic of Korea; okgksmsla@dgu.ac.kr (P.K.); rlathdus4152@gmail.com (S.Y.K.); shijiayu872@gmail.com (J.S.); 2 College of Pharmacy and Research Institute of Pharmaceutical Sciences, Seoul National University, 1 Gwanak-ro, Gwanak-gu, Seoul 08826, Republic of Korea

**Keywords:** health functional foods, Individually Recognized Functional Ingredients (IRFIs), regulatory science, Korean Ministry of Food and Drug Safety (MFDS)

## Abstract

**Background/Objectives:** The global health functional food (HFF) market is expanding rapidly, driven by increasing consumer interest in preventive healthcare and evidence-based nutrition. The Republic of Korea has established a systematic regulatory framework for HFFs, through the Individually Recognized Functional Ingredient (IRFI) system introduced in 2004. Designed to accommodate innovative physiologically active ingredients beyond standardized categories, the IRFI system is increasingly discussed as a regulatory model for evidence-based functional foods. This study examines the IRFI system within a comparative regulatory context and evaluates its implications for drug–dietary supplement interactions (DDSIs). **Methods**: Functional categories were defined according to guidelines issued by the Ministry of Food and Drug Safety (MFDS). Data on Korean IRFIs were obtained from the Food Safety Korea database. A literature search was conducted in PubMed using regulatory keywords which identified through Google searches. Regulatory frameworks in the United States, European Union, Japan, and China were comparatively analyzed. DDSIs were reviewed based on MFDS-approved IRFIs and the relevant literature. **Results**: The IRFI represents a hybrid regulatory model that combines rigorous pre-market scientific evaluation, including GLP-compliant safety testing and human clinical evidence, with regulatory incentives such as expedited review and temporary market exclusivity. Compared with post-market-oriented systems in the United States and Japan, the stringent authorization framework in the European Union, and the dual-track health food system in Chinese, IRFI integrates clinical evidence requirements within a structured pre-market approval process. **Conclusions**: The IRFI framework establishes a comparatively stringent evidentiary standard for functional foods while providing a structured basis for evaluating potential DDSIs. Its applicability depends on alignment and mutual recognition of scientific and clinical evaluation criteria across regulatory jurisdictions. DDSIs were reviewed based on MFDS-approved IRFIs and the relevant literature.

## 1. Introduction

Global interest in **Health Functional Foods (HFFs)** has increased markedly in recent years, driven by growing awareness of preventive healthcare, the rising burden of chronic diseases, and demographic transitions toward aging populations [1,2]. Alongside industrial development, consumers’ interest in overall well-being and self-care has intensified, leading to heightened demand for products that support health maintenance and disease risk reduction. In response, countries worldwide are strengthening regulatory policies and frameworks to enhance consumer trust in health functional foods, contributing to the rapid expansion of the global HFF market [1]. Within this global context, East Asia has emerged as a particularly influential region in the HFF sector. Among East Asian countries, the Republic of Korea is frequently highlighted as a model for rapid yet well-structured industry development, supported by a robust regulatory infrastructure, sustained market growth, and high levels of consumer trust [3,4]. According to the Korea Health Functional Food Association (KHFA), a government-recognized industry association, the Korean HFF market exceeds 5 trillion KRW of 2024, underscoring its increasing economic significance and public health relevance [5,6].

Historically, the Korean HFF market relied predominantly on **standardized ingredients (SIs)** defined within the Health Functional Food Code. These pre-approved ingredients allow manufacturers to enter the market with minimal regulatory delay and relatively low barriers to entry, thereby facilitating broad access and rapid industry expansion. However, the SI-based system also imposed inherent limitations, including restricted opportunities for product differentiation, limited incentives for scientific innovation, and insufficient flexibility to accommodate increasingly diverse consumer needs. As a result, many HFF products exhibited functional redundancy across manufacturers. For example, *Panax ginseng* C.A. Meyer, as a standardized functional ingredient, is approved for multiple functional claims including immune enhancement, fatigue improvement, bone health, and liver health, illustrating functional redundancy in which a single ingredient encompasses diverse yet overlapping physiological functions [7].

To address these challenges in Korea, the Ministry of Food and Drug Safety (MFDS) introduced the **Individually Recognized Functional Ingredient (IRFI)** system in 2004. This science-based regulatory pathway enables the independent evaluation and approval of novel bioactive ingredients not included among existing standardized materials [4,8,9]. The IRFI system was designed to overcome the limitations of SI-based regulation by requiring robust scientific evidence for both safety and functional efficacy, thereby strengthening consumer confidence through government-led pre-market assessment grounded in clinical research [8,10]. Under the IRFI framework, human intervention studies are generally required to substantiate functional claims; however, the specific study design does not uniformly mandate randomized controlled trials (RCTs) and may vary depending on the functional category, target population, and the strength of existing scientific evidence, as determined by MFDS evaluation guidelines [4,5,8,9].

Importantly the IRFI framework reflects a conceptual transition from generalized nutritional support toward precision-oriented functional modulation. In this context, “precision-oriented functional modulation” refers to population-level targeting of specific physiological functions based on shared risk profiles, mechanistic evidence, and clinically validated endpoints, rather than individualized genetic or microbiome-based customization, which remains outside the current regulatory scope. The system supports function-specific interventions for defined population groups, including older adults, individuals with chronic diseases, and other high-risk populations. This regulatory approach has facilitated advances across a broad spectrum of functional domains, including immune modulation, blood pressure and glucose regulation, sleep quality, cognitive performance, and metabolic balance [8,10,11]. Moreover, the requirement for human clinical evidence provides a structured foundation for the future integration of pharmacokinetic, pharmacodynamic, and interaction-based evaluations, particularly in populations with concurrent medication use.

This review provides a comprehensive evaluation of the Korean IRFI system by examining (1) its legal and regulatory foundations, (2) the functional classification framework, (3) the current approval landscape, and (4) its implications for scientific innovation and public health. In addition, comparative analyses with major international regulatory models—including those of the United States, the European Union, Japan, and China—are presented to contextualize Korea’s status within the global functional food regulatory landscape. Furthermore, as the clinical use of HFFs expands, systematic consideration of pharmacokinetic and pharmacodynamic characteristics, as well as potential drug–nutrient interactions, is becoming increasingly important to ensure both safety and functional reliability in real-world settings.

## 2. Methodology

A functional classification framework was established based on evaluation guidelines issued by Korean MFDS [12,13]. Information on Korean IRFIs was obtained from the Food Safety Korea database, while international regulatory information was collected through PubMed and Google searches.

A literature search was conducted in PubMed covering publications from 1990 to 2025 using the terms “health functional food,” “regulatory system,” and “functional ingredient approval,” combined with country-specific keywords. Regulatory frameworks in the United States [U.S. Food and Drug Administration (FDA)], European Union [European Medicines Agency (EMA) and European Food Safety Authority (EFSA)], Japan [Consumer Affairs Agency (CAA)], and China [National Medical Products Administration (NMPA) or the State Administration for Market Regulation (SAMR)] were comparatively analyzed to assess functional equivalence with the Korean system.

Functional categories were organized according to MFDS evaluation guidelines [12,13]. IRFIs were mapped to their corresponding functional categories, and the relevant literature was reviewed to assess evidence of functional efficacy, safety, and potential drug–dietary supplement interactions (DDSIs). Comparative data presented in Tables were extracted based on direct relevance to the functional claims investigated.

## 3. Functional Classification System of IRFIs in Korea

The MFDS classifies the functionalities of HFFs into 41 scientifically defined categories, providing a structured framework for the evaluation of functional claims and regulatory decision-making [14]. Within this regulatory architecture, IRFIs are subject to a stringent, evidence-based review process that is distinct from the streamlined regulatory pathway applied to SIs pre-listed in the Health Functional Food Code [4,14,15]. Whereas SIs are broadly accessible to all manufacturers and can be used without additional pre-market evaluation, IRFIs are proprietary ingredients that require sponsor-generated scientific dossiers. These dossiers must include comprehensive safety assessments, human intervention trials, and detailed specifications to substantiate functional efficacy and ensure consumer’s safety [16,17]. As of December 2025, a total of 489 HFFs, comprising 96 SIs and 393 IRFIs, have been approved under the Korean regulatory framework (Food Safety Korea database, Ministry of Food and Drug Safety). This expansion reflects both the rapid growth of the Korean HFF industry and the increasing scientific diversification of functional ingredient development [10,15,16,17].

### 3.1. Definition of HFFs and Regulatory Structure of Functional Ingredients

In the Republic of Korea, **HFFs** are defined under the Health Functional Foods Act and Article 3 of the Health Functional Food Code as foods manufactured or processed using ingredients with verified health functionality beyond basic nutrition [18,19]. Functionality refers to scientifically substantiated effects that contribute to the maintenance or improvement of health, modulation of physiological functions, or reduction in disease risk. This definition reflects rigorous Korean application of an evidence-based evaluation system and clearly distinguishes HFFs from conventional foods and pharmaceuticals through legally defined functional and regulatory criteria [15,18,19]. Accordingly, the development and commercialization of HFFs are governed by a comprehensive regulatory framework encompassing both pre-market authorization and post-market surveillance to ensure safety, functional reliability, and consumer protection [17]. The MFDS evaluates functional ingredients based on safety, functional efficacy, manufacturing quality, and specification standards, applying a dual regulatory structure consisting of IRFIs (Table 1).

#### 3.1.1. Standardized Ingredients (SIs)

SIs are functional materials formally listed in the Health Functional Food Code based on accumulated scientific evidence demonstrating safety, efficacy, and manufacturing consistency. Because these ingredients possess predefined specifications and validated functional claims, manufacturers may use them without submitting additional scientific data to the MFDS, provided that established quality control and manufacturing standards are met. Although standardized ingredients are pre-listed in the Health Functional Food Code, they may still be subject to post-market safety monitoring and reassessment in response to emerging safety data, adverse event reports, or regulatory review by the MFDS [24]. As of December 2025, the Health Functional Food Code includes 96 SIs, comprising 28 vitamins and minerals and 68 functional ingredients, supporting a broad range of commonly used health functionalities [3,8,9,13,16,17,25]. This regulatory pathway enhances market accessibility and significantly reduces development costs by allowing the use of well-characterized ingredients without ingredient-specific regulatory review.

#### 3.1.2. Individually Recognized Functional Ingredients (IRFIs)

**IRFIs** refer to novel or previously unlisted functional materials that are not included among SIs. Because these ingredients lack predefined specifications or established histories of functional use, applicants seeking IRFI approval must submit a comprehensive scientific dossier to the MFDS for evaluation [3,4,8,9,12,13,16,17,23].

The required dossier includes:

- **GLP-compliant toxicological studies**, determined by risk-based assessment, may include acute, sub-chronic or chronic toxicity, reproductive and developmental toxicity, and genotoxicity. The specific toxicological endpoints are not uniformly mandated for all ingredients; rather, the scope and depth of required studies are determined based on ingredient characteristics, intended use levels, duration of exposure, and the availability of existing safety data, in accordance with MFDS evaluation guidelines;
- **RCTs** demonstrating statistically and clinically meaningful functional efficacy;
- **Compositional characterization**, including purity profiles, active compound identification, and potential contaminants;
- **Stability data and manufacturing information**, including production processes, quality control procedures, and specification standards to ensure lot-to-lot consistency.

Upon successful evaluation, the ingredient is approved as an IRFI and granted exclusive commercial use by the applicant company for a period of six years. Nevertheless, concerns have been raised that extended exclusivity periods may limit market competition and delay broader industry participation, prompting ongoing discussions regarding the optimal duration and scope of exclusivity within the IRFI framework [26]. This exclusivity provision serves as an important incentive for investment in high-quality research and development by protecting the proprietary value of novel functional materials.

### 3.2. Classification and Current Status of the 41 Functional Categories (As of December 2025)

The MFDS currently classifies HFFs into 41 functional categories, providing a structured regulatory framework that ensures scientific rigor, consumer safety, and regulatory transparency. Functional categories are revised or newly established through MFDS regulatory review processes that incorporate emerging scientific evidence, expert evaluation, updates to functional assessment guidelines, and reassessment of public health needs. This classification system applies defined evidentiary thresholds for functional recognition while allowing flexibility to accommodate evolving consumer health demands. As of December 2025, more than 393 IRFIs have been approved across these categories, illustrating the breadth of the regulatory system’s capacity to evaluate diverse bioactive materials [16]. Compared with 2023, seven additional functional categories have been introduced, including more specific functional items such as hair and gum health [27].

The functional classification framework is implemented through MFDS guidance documents that specify evidence requirements for functional recognition, including target populations, biomarker selection, mechanistic justification, acceptable study designs, and minimum thresholds for clinical efficacy. Within this framework, each functional category encompasses a defined set of scientifically validated ingredients. For example, the blood glucose regulation category includes ingredients such as mulberry leaf extract and banaba extract, which have demonstrated effects on postprandial glucose metabolism, while the immune enhancement category includes probiotic **strains (e.g., *Lactobacillus*** species), β-glucans, and polysaccharide-rich botanical extracts supported by evidence of immunomodulatory activity [8,28,29,30,31,32,33,34,35,36,37,38].

To achieve functional recognition, ingredients must demonstrate reproducible positive effects on relevant physiological indicators—such as glycemic control, immune responsiveness, or sleep quality—based on accumulated scientific evidence. Although HFFs are distinct from pharmaceuticals, claimed physiological effects must be supported by evidence extending beyond single studies, including mechanistic validation and repeated human intervention trials. Acceptable evidence under the IRFI framework may include multiple randomized controlled trials (RCTs), systematic reviews, meta-analyses, or combinations thereof. While meta-analyses are encouraged to strengthen assessments of reproducibility when sufficient high-quality data are available, they are not mandatory provided that consistent evidence from multiple well-designed human studies can be demonstrated [39]. Establishment of an effective daily intake similarly requires reproducible findings from rigorously designed studies [17].

For IRFI approval within a given functional category, applicants must submit scientific evidence aligned with the targeted physiological effect, typically including human intervention studies, validated functional biomarkers, and mechanistic data. Evidence requirements vary across functional areas such as cognitive function, metabolic regulation, and immune modulation, which generally require more rigorous study designs, shaping research strategies across the functional ingredient industry [8,11,19,29].

When the IRFI system was introduced in 2004, only nine functional categories were recognized, primarily focusing on cardiometabolic and age-related functions [35]. Over time, the classification system expanded to include domains such as sensitive skin improvement, muscle strength enhancement, gut microbiome modulation, menopausal symptom alleviation, and sleep quality improvement, reflecting broader societal trends including population aging and increasing chronic disease prevalence [8,9,10,11,19]. Despite this expansion, functional labeling remains strictly regulated to prevent overlap with pharmaceutical claims; HFFs are limited to claims related to the maintenance or improvement of normal physiological functions [9,19,29].

Table 2 summarizes representative IRFIs across the 41 functional categories, illustrating functional distribution patterns, areas of concentrated regulatory or industrial activity, and emerging directions for research and regulatory refinement.

## 4. Background of the IRFI System in Korea and Its Current Implications

The establishment of the IRFI system in the Republic of Korea represents a strategic regulatory response to evolving scientific, industrial, and public health demands. As the HFF market expanded, consumer expectations increasingly shifted toward personalized, evidence-based functional solutions. In response, regulators moved beyond reliance on pre-notified SIs and introduced an independent evaluation and approval pathway capable of assessing novel bioactive substances with greater scientific precision. The IRFI system was designed to address the limitations of conventional regulatory approaches, particularly the difficulty of evaluating new functional ingredients lacking predefined specifications or historical use data. By incorporating independent, science-based verification processes, the system enhanced safety assurance while supporting innovation. Today, the IRFI framework is widely recognized as a key driver of the Korean functional food innovation ecosystem, fostering advanced research, targeted product development, and global competitiveness.

### 4.1. Regulatory Background and Market Drivers

The development of the IRFI system was shaped by multiple structural and market-level forces. First, rising consumer interest in personalized health management created demand for more refined functional claims and greater ingredient diversity within existing functional categories. Consumers increasingly sought products tailored to specific health concerns—such as metabolic regulation, immune resilience, sleep quality, and age-related physiological decline—which could not be adequately addressed by SIs alone [45,46].

Second, national policies emphasizing high value-added innovation in the food and biotechnology sectors encouraged the development of proprietary functional materials. The IRFI framework supports such innovation by granting temporary market exclusivity for approved ingredients, thereby promoting fair competition and incentivizing substantial investment in research and development [47].

Third, the rapid global expansion of the functional food market underscored the need for regulatory systems capable of rigorously evaluating novel bioactive components [48,49]. Early regulatory approaches in Korea relied heavily on SIs, which facilitated rapid product development but limited opportunities for differentiation. As scientific advances introduced new classes of functional materials—including botanical extracts, probiotics, peptides, and bioactive phytochemicals—the need for an individualized evaluation system became increasingly apparent. The IRFI system emerged as a natural regulatory evolution to accommodate these advances [47,50].

Under the IRFI pathway, applicants are required to submit comprehensive scientific dossiers, including randomized controlled human trials, GLP-compliant toxicological data, validated functional biomarkers, and detailed manufacturing specifications [8,47]. Upon approval, ingredients are granted exclusive commercial use for a period of six years, after which they may be reclassified as SIs [51]. This exclusivity mechanism not only rewards innovation but also promotes sustained investment in functional ingredient development. IRFI approvals typically target clearly defined physiological outcomes—such as improvements in sleep architecture, alleviation of menopausal symptoms, enhancement of immune function, or modulation of metabolic pathways—rather than broad, nonspecific health claims [4,8,11,15,52,53,54]. This reflects a broader transition from general nutritional supplementation toward precision-oriented functional modulation, positioning IRFIs at the intersection of food science, biotechnology, and personalized wellness.

From an industrial perspective, IRFIs may offer strategic advantages in premium product segments, hospital-linked distribution channels, and export-oriented commercialization. MFDS-approved scientific evidence can also facilitate regulatory access in certain foreign jurisdictions, including the U.S. New Dietary Ingredient (NDI) framework, the EU Novel Food and health claim systems, and Chinese health food registration pathway. As a result, several Korean IRFIs—particularly probiotic strains, functional peptides, and botanically derived ingredients supported by human clinical data—have been positioned as higher value-added products in global markets [4,8,11,15,52,53,54].

### 4.2. Trends in the Expansion of Functional Categories and Their Implications

When first introduced in 2004, the Korean HFF regulatory framework encompassed only nine functional categories, primarily focused on cardiometabolic health, cognitive function, and joint-related outcomes. Over time, accumulating scientific evidence, demographic transitions, and increased health awareness drove substantial diversification. By 2025, the MFDS recognized 41 distinct functional categories, reflecting a marked expansion in both functional scope and targeted physiological domains [10,11,49,50,55,56,57].

Newly established categories—such as microbiome modulation, stress regulation, menopausal care, sensitive skin improvement, muscle strength maintenance, and sleep quality enhancement—illustrate a transition toward targeted functional interventions aligned with evolving public health priorities. This qualitative shift closely parallels demographic aging trends; Korea has already entered a super-aged society, with approximately 21% of the population projected to be 65 years of age or older by 2025, thereby contributing to increased regulatory and scientific attention on functional categories addressing age-associated physiological changes [58].

As functional claims have become more granular, the evidentiary standards required for functional recognition have intensified, necessitating deeper mechanistic understanding, refined biomarker selection, and more sophisticated clinical study designs. In response, MFDS guidance documents have progressively incorporated more stringent scientific evaluation criteria, reinforcing expectations for reproducibility and clinical relevance. Industry strategies have concurrently shifted away from broad-spectrum claims toward population- and lifecycle-oriented functionalities targeting older adults, peri-menopausal women, individuals with metabolic disorders, or consumers seeking microbiome-related benefits.

At the same time, refinement of functional categories has facilitated closer alignment with international regulatory frameworks, particularly the EFSA’s health claim evaluation standards, thereby strengthening scientific rigor and enhancing the international credibility of Korean IRFIs. The expanded classification system has also promoted academic–industry collaboration and multidisciplinary research integrating food science, pharmacology, microbiology, and clinical nutrition, while supporting a shift toward greater precision and lifecycle-focused functional development. Collectively, these trends indicate that the evolution of Korea’s functional classification system has driven both quantitative growth and qualitative sophistication within the HFF industry, while continued progress will require sustained regulatory refinement and closer alignment with global scientific and regulatory landscapes [10,11,52,53,55,56,57].

## 5. Comparative Analysis of IRFI Systems Across Major Countries

The IRFI system of the Republic of Korea is distinguished by its rigorous scientific evaluation, structured approval process, and unique market exclusivity provisions. To contextualize the Korean regulatory approach within the global landscape, it is essential to compare it with functional ingredient regulatory systems in major jurisdictions, including the United States, the European Union, Japan, and China. These systems differ substantially in regulatory philosophy, evidentiary requirements, approval pathways, and the extent of governmental oversight, offering valuable insights into potential harmonization strategies and future policy development.

Collectively, these comparisons position the Korean IRFI system as a hybrid regulatory model, balancing scientific rigor comparable to the European framework with innovation incentives that exceed those of most global counterparts. In particular, the exclusivity provision lowers the financial risk associated with early-stage research investment and enables the development of high-quality functional ingredients within a relatively short timeframe. At the same time, the mandatory requirement for robust human clinical evidence ensures scientific credibility, establishing the Korean system as both industrially strategic and scientifically grounded. Continued alignment with international regulatory standards will be critical for facilitating global market access and the regulatory interoperability of Korean IRFIs [4,8,59,60] (Table 3).

### 5.1. United States: Dietary Supplement Health and Education Act (DSHEA) and the NDI System

In the United States, dietary supplements are regulated primarily under the DSHEA of 1994, which classifies dietary supplements as a category of conventional foods. Under the DSHEA, most dietary supplements may be marketed without pre-market approval, reflecting a regulatory philosophy that emphasizes market access and post-market oversight. However, dietary ingredients not marketed prior to 1994 are classified as NDIs and require pre-market notification to the U.S. FDA [70,71]. Despite this regulatory requirement, the NDI system has significant limitations: the FDA has estimated that approximately 3400 products containing NDIs have been marketed without required notification [72], and NDI submissions frequently face rejection due to unclear safety expectations, while post-market oversight remains largely reactive [73,74].

The NDI pathway shares conceptual similarities with the Korean IRFI system in that both require submission of scientific data. However, the evidentiary thresholds differ substantially. Under the U.S. framework, human clinical trials are not mandatory, and manufacturers are required only to demonstrate that an ingredient is reasonably expected to be safe, rather than to substantiate functional efficacy [70,74,75]. Consequently, the evidentiary standard is considerably lower than that of the Korean IRFI system, which requires RCTs to support functional claims.

Overall, the U.S. dietary supplement regulatory model allows ingredients with limited clinical validation to circulate prior to comprehensive evaluation, while offering advantages in regulatory efficiency and market accessibility, particularly for small and medium-sized enterprises [70,74,75].

### 5.2. European Union: Novel Food Regulation and Health Claim Authorization

The European Union maintains one of the most stringent regulatory environments for functional ingredients through the Novel Food Regulation and the Health Claim Regulation (EC No. 1924/2006). Under the Novel Food framework, ingredients without a documented history of consumption prior to 1997 are required to undergo comprehensive safety evaluation by the EFSA [76,77]. Although the EFSA’s scientific assessments result in approval rates of approximately 87%, the authorization process is procedurally demanding, with an average timeline of 2.56 years from submission to publication of safety opinions and multiple additional data requests per application [64]. In practice, a substantial proportion of submitted health claim applications are ultimately rejected by the EFSA, highlighting the high evidentiary threshold required for authorization.

Authorization of health claims in the EU is subject to particularly rigorous scientific substantiation. Evidence is primarily required from well-designed human RCTs, whereas in vitro studies, animal experiments, or mechanistic hypotheses alone are considered insufficient. Applicants must demonstrate validated biomarkers, dose–response relationships, and clearly defined physiological endpoints. Consequently, the EU’s evidentiary standards for functional claims are comparable to those of the Korean IRFI system in terms of scientific rigor [76,77,78].

A key distinction from the Korean model lies in the absence of exclusivity provisions. Once a Novel Food or health claim is authorized, approval becomes publicly accessible to all manufacturers. While this approach promotes transparency, consumer protection, and equitable market access, it may limit incentives for individual companies to invest in costly clinical research and prolonged regulatory procedures. Overall, the EU framework prioritizes precaution, public health protection, and scientific rigor over commercial incentives, reflecting a strongly science-centered regulatory philosophy [76,77,78].

### 5.3. Japan: Foods with Function Claims (FFCs) and CAA

The Japanese government introduced the FFC system in 2015 to promote industry autonomy and accelerate the commercialization of functional foods. Under this framework, companies may notify the CAA of functional claims based on either human clinical trials or systematic literature reviews, without undergoing formal governmental scientific evaluation [60,79,80,81,82,83,84].

This approach contrasts sharply with the Korean IRFI system, which mandates human clinical trials and centralized regulatory review. The FFC system enables rapid market entry—often within approximately 60 days of notification—thereby reducing regulatory burden and fostering innovation. However, the absence of pre-market scientific verification has raised concerns regarding heterogeneity in evidence quality, inconsistencies in study design, and variability in biomarker validity across products [60,79,80,81,82,83,84]. Importantly, independent methodological evaluations of the FFC system have identified substantial weaknesses in the quality of systematic reviews supporting notified claims. Studies assessing FFC-related submissions reported that systematic reviews filed after the introduction of the FFC system exhibited significantly poorer methodological and reporting quality than earlier reviews, including inadequate descriptions of search strategies, study selection, data extraction, risk-of-bias assessment, and publication bias evaluation [85].

Although the FFC system has enhanced industry flexibility, its reliance on post-market monitoring places greater responsibility on manufacturers to ensure scientific integrity. Regulatory reviews and academic analyses have therefore emphasized the need for strengthened post-market surveillance and transparency mechanisms to maintain consumer trust [60,65,79,80,81,82,83,84,86,87]. Overall, the Japanese regulatory model promotes innovation through deregulation while requiring robust post-market oversight to safeguard consumers.

### 5.4. China: Registration and Health Food Approval System

Chinese government regulates HFFs under the Health Foods category through a dual-track system established in 2016, consisting of a registration pathway for novel or higher-risk products and a notification (filing) pathway for products using pre-approved ingredients and claims [88]. These pathways differ substantially in evidentiary requirements, regulatory oversight, and time to market.

Under the registration pathway, novel functional ingredients or products making higher-level functional claims must undergo comprehensive evaluation by the NMPA or the SAMR, including submission of safety and efficacy data such as human intervention studies or animal experiments [89,90,91]. In contrast, the notification pathway allows expedited market entry—often within days—for products formulated with approved raw materials and claims listed in the HFF list, without formal pre-market scientific review [92]. The Chinese government strictly regulates functional claim wording and prohibits claims implying disease prevention or treatment, maintaining a clear boundary between foods and pharmaceuticals [93]. Post-approval obligations, including periodic safety verification and documentation updates, further reinforce regulatory accountability and consumer protection [90].

Overall, the Chinese Health Foods framework integrates centralized regulatory oversight with national industrial and public health strategies. Compared with the Korean IRFI system, the Chinese model emphasizes policy coordination and tiered regulation based on product risk and innovation level [89,90,91,94,95,96].

## 6. Drug–Dietary Supplement Interactions (DDSIs) in IRFIs

The increasing concomitant use of **HFFs** and prescription medications has heightened attention on **DDSIs**. Although HFFs are regulated as foods rather than medicinal products, many **IRFIs** possess bioactive properties that can modulate metabolic enzymes, drug transporters, and physiologically relevant signaling pathways. When co-administered with drugs, such effects may alter pharmacokinetics (PK) and/or pharmacodynamics (PD), potentially increasing toxicity risk or reducing therapeutic effectiveness. As IRFIs increasingly resemble pharmacological agents in potency and mechanism, systematic assessment of DDSI risk has become an important clinical and regulatory consideration [97,98].

### 6.1. Potential Mechanisms for DDSIs

DDSIs may occur through mechanisms analogous to classical drug–drug interactions. A major pathway involves modulation of drug-metabolizing enzymes. Certain botanical or microbially derived functional ingredients can inhibit or induce cytochrome P450 (CYP) enzymes, thereby changing the metabolic clearance of co-administered medications. Well-known examples include induction of CYP3A4 by Hypericum perforatum (St. John’s wort) and inhibition of CYP2C19 by specific flavonoids [99,100].

Transporter-mediated interactions represent another key mechanism. Functional ingredients may inhibit or influence the activity of transporters such as P-glycoprotein (P-gp), breast cancer resistance protein (BCRP), and organic anion transporting polypeptides (OATPs), thereby affecting drug absorption, distribution, and tissue exposure. DDSIs may also arise through overlapping physiological or PD pathways. Ingredients intended to modulate glycemic control, blood pressure, lipid metabolism, coagulation, or neurotransmission may produce additive or synergistic effects when combined with therapeutic agents. Such interactions can increase the risk of clinically relevant outcomes, including hypoglycemia, hypotension, excessive anticoagulation, or central nervous system adverse effects [98,101,102,103].

With ongoing advances in precision nutrition and pharmacogenomics, predicting and managing DDSIs is becoming increasingly important for both safe functional ingredient development and real-world clinical use. Accordingly, international regulatory authorities have placed growing emphasis on interaction-related risk characterization when warranted. For example, risk communication (including precautionary labeling) may be expected for ingredients with well-documented interaction potential, and regulatory dossiers for certain health claims or novel food applications may require consideration of interaction risk depending on the ingredient’s mechanism and intended use [58,104,105] (Table 4).

### 6.2. Regulatory Approaches to Interaction Assessment for IRIFs

Although the United States, the European Union, and the Republic of Korea all recognize the potential for DDSIs, their regulatory approaches differ in terms of mandatory requirements, evidentiary thresholds, and the balance between pre-market expectations and post-market oversight. Overall, regulatory attention on interaction assessment is increasing, yet substantial variation persists across jurisdictions regarding standardized pre-market evaluation and post-market adverse effect monitoring [8,58,59,105,126].

In general, the U.S. approach is primarily safety-focused and post-market oriented, with interaction considerations addressed when a risk signal is anticipated from existing evidence. The EU framework is comparatively more stringent and may integrate interaction considerations into pre-market scientific substantiation for health claims and novel foods. Korean MFDS adopts a hybrid approach in which interaction-related information can be included within safety dossiers, although a formalized, standalone DDSIs testing requirement is not consistently mandated [8,58,59,105,126].

As functional ingredients become more pharmacologically active, the need for harmonized frameworks and structured DDSIs assessment methodologies has been increasingly recognized. Regulatory science is gradually moving toward mechanistic evaluations, population-specific risk stratification, and integration of real-world evidence through pharmacovigilance systems capable of identifying emerging interaction signals [8,58,59,105,126] (Table 5).

#### 6.2.1. United States FDA

In the United States, dietary supplements are regulated under DSHEA from 1994, which allows market entry without pre-market approval and does not generally require animal studies or clinical trials. Formal DDSI testing is not mandated for most products. However, under the NDI notification pathway, manufacturers must demonstrate that a novel ingredient is “reasonably expected to be safe,” and interaction considerations may be included when available evidence suggests effects on CYP enzymes, drug transporters, or clinically sensitive physiological pathways [75,104,126].

FDA guidance for drug development—particularly documents addressing CYP- and transporter-mediated interactions—often informs general scientific expectations regarding interaction risk characterization, although this guidance is not legally binding for dietary supplements. For supplements with well-established interaction *concerns (e.g., St.* John’s wort, Ginkgo biloba, red yeast rice), precautionary risk communication and labeling are commonly emphasized. In practice, post-market surveillance, including adverse event reporting, remains the principal mechanism for identifying and managing DDSI risks in the U.S. context [75,104,126,127].

#### 6.2.2. European Union: The European Medicines Agency (EMA) and EFSA

The European Union applies a more stringent pre-market scientific framework, particularly through EFSA evaluations under Regulation (EC) No. 1924/2006. Compared with the U.S. system, the EU typically requires robust substantiation of safety and efficacy for certain regulated categories, and interaction considerations may be addressed when an ingredient is expected to affect metabolic pathways or PD domains relevant to drug therapy.

Dossiers submitted to the EFSA are expected to include detailed characterization of composition, proposed mechanisms of action, and relevant safety information, which may encompass interaction-related evidence when applicable. Human RCTs frequently form the evidentiary core for substantiating functional claims, supported by mechanistic and toxicological data. If an ingredient is known or suspected to modulate CYP enzymes, drug transporters, hemostasis, or cardiometabolic markers, clear documentation of these effects and their potential relevance to co-administration scenarios may be required [58,59,77].

Although the EMA primarily regulates medicinal products (including certain herbal medicinal products), its scientific guidance on pharmacokinetic interactions can provide supplementary context for interaction risk assessment in related domains. Overall, the EU framework prioritizes transparency and consumer protection through rigorous scientific evaluation, without a commercial exclusivity mechanism for approved claims or ingredients [128].

#### 6.2.3. Republic of Korea: KFDA (MFDS)

In Korea, the HFFs Act establishes a dual regulatory structure comprising standardized ingredients and IRFIs. A distinctive feature is the **six-year exclusivity** granted to newly approved IRFIs, which incentivizes R&D and supports innovation. While the Korean government does not currently mandate a standalone pre-market DDSI testing package, interaction-related considerations may be incorporated within broader safety evaluations.

Applicants are expected to submit available evidence relevant to ADME characteristics, including known or predicted effects on CYP enzymes, major *transporters (e.g., P-gp*, BCRP), and any interaction signals reported in the literature. In addition, clinical study designs should justify participant selection, including considerations of concomitant medication use when it may influence safety or functional outcomes—particularly for categories related to blood glucose or blood pressure regulation [98,99,100,101,102,129].

Post-market surveillance remains central for detecting emerging DDSI signals. HFFs are subject to adverse event reporting, and products suspected of clinically meaningful interactions may undergo re-evaluation or require modifications to labeling, intake instructions, or authorized claims. Recent policy discussions have highlighted the need for stronger, more standardized integration of DDSI considerations into IRFI evaluations, especially for ingredients that overlap mechanistically with therapeutic drug classes or influence clinically sensitive physiological pathways [8,130,131,132].

Priority areas for regulatory enhancement include systematic evaluation of enzyme- and transporter-mediated interactions, risk stratification for high-risk populations, targeted review for categories with inherently high interaction potential, and integration of real-world evidence *sources (e.g., electronic* medical records) to support proactive pharmacovigilance [133,134,135,136].

## 7. Limitations and Future Perspectives

The Korean IRFI system exhibits distinctive regulatory features alongside several structural and scientific challenges. These limitations arise from the inherent complexities of functional ingredient evaluation, evolving scientific methodologies, and changing consumer health expectations. Addressing these gaps represents an ongoing priority for regulatory refinement.

A major limitation concerns the variability in the quality and comparability of sponsor-submitted evidence. Although the Korean IRFI pathway requires human intervention trials and GLP-compliant safety studies, submitted dossiers can differ substantially in study design, statistical power, biomarker selection, and clinical relevance. Such heterogeneity complicates cross-ingredient comparisons and constrains the development of standardized methodological benchmarks. Moreover, because clinical evidence is typically sponsor-generated, risks related to publication bias, underpowered studies, limited follow-up, and insufficient diversity of study populations may reduce generalizability and reproducibility of functional claims.

A second challenge arises from the increasing pharmacological sophistication of IRFIs. As functional ingredients increasingly target sensitive physiological domains—such as glycemic regulation, blood pressure control, neuronal pathways, and immune modulation—the boundary between foods and pharmaceuticals becomes progressively narrower. However, Korea, like most jurisdictions, lacks a dedicated framework for systematic evaluation of DDSIs. Interaction-related considerations are currently incorporated indirectly within safety evaluations, and standardized protocols are not consistently established for assessing enzyme- or transporter-mediated interactions, PK/PD alterations, or additive pharmacodynamic effects. These gaps may limit safety assessments, particularly in high-risk populations such as the elderly and those with chronic diseases taking multiple medications.

From a regulatory systems perspective, international harmonization is *lacking (e.g., the* U.S. FDA, EFSA, and Chinese SAMR/NMPA). These differences pose regulatory obstacles to mutual recognition between Korean IRFIs and market entry by multinational companies. Furthermore, the six-year exclusivity regime could limit data sharing and accumulated knowledge. Several strategic directions for future improvements to the IRFI system could be considered. First, establishing **standardized methodological guidance** for human intervention trials—including harmonized endpoints, core biomarker panels, minimum sample-size expectations, and validated surrogate markers—could enhance evidence consistency and regulatory predictability. Second, **structured DDSI risks assessment** into IRFI dossiers—particularly for functionalities with high interaction potential—could strengthen safety oversight. A tiered approach might include in vitro CYP inhibition/induction testing, transporter assays, physiologically based pharmacokinetic (PBPK) modeling, and targeted clinical interaction studies when warranted.

Third, integrating **real-world data (RWD)** sources—such as electronic medical records, national health insurance databases, product consumption registries, and pharmacovigilance platforms—might facilitate continuous safety monitoring beyond conventional clinical trials. Such infrastructure could potentially support earlier detection of adverse events, long-term monitoring, and identification of population-specific benefits or risks not captured in conventional clinical trials.

Finally, convergence between IRFIs and emerging fields, including precision nutrition, genomics, gut microbiome science, digital health technologies, and AI-enabled ingredient discovery presents potential opportunities for development. These approaches could support population-specific or genotype-informed functional ingredients, enable dynamic personalization of intake recommendations, and facilitate evidence generation through digital phenotyping and real-time biomarker monitoring. Global trends toward clinically integrated nutritional interventions highlight areas for potential regulatory consideration.

In summary, the Korean IRFI system provides a robust foundation for evidence-based functional ingredient development. Further progress will require (i) strengthening scientific credibility through methodological standardization, (ii) modernizing safety assessment—potentially incorporating PBPK modeling as part of a tiered evaluation strategy alongside conventional approaches, and (iii) enhancing international interoperability through regulatory harmonization and cross-border cooperation in precision-oriented functional foods.

## 8. Conclusions

Comparative analysis of major regulatory frameworks in the United States, European Union, Japan, and China indicates that the Korean IRFI system is characterized by a regulatory structure that combines pre-market scientific evaluation with industry-oriented incentive mechanisms [59,60]. While the EU framework prioritizes stringent pre-market substantiation and transparency, and the U.S. and Japanese systems emphasize market flexibility and post-market responsibility, the Korean approach integrates clinical evidence requirements with time-limited exclusivity to support the development of novel functional ingredients. Future refinement of the IRFI system will require closer alignment with international standards, particularly in the assessment of drug–dietary supplement interactions, as well as improved mechanisms for cross-jurisdictional recognition of scientific evidence. As functional ingredients increasingly target disease-adjacent physiological domains, maintaining clear regulatory boundaries between foods and pharmaceuticals will be essential to ensure consumer protection while supporting scientific and industrial advancement [128,137].

## Figures and Tables

**Table 1:** Comparison between SIs and IRFIs.

Category	SIs	IRFIs	Refs.
Definition	Ingredients listed in the official Health Functional Food Code	Ingredients approved through individual scientific review	[20,21]
Approval process	No separate review required	Requires submission of data such as human intervention studies followed by official evaluation	[12,20,21]
Users	Available for use by any manufacturer	Use restricted to the company that received approval	[20,21,22]
Conversion to SIs	Not applicable	Possible after 6 years	[13]
Examples	Vitamin C, Red ginseng, EPA/DHA	Chicken sternal cartilage powder, Krill oil-containing complex, *Lactobacillus plantarum* TWK10 probiotics	[13,23]

**Table 2:** Examples of MFDS-approved IRFIs by functional category (2025) [7,23,40,41,42,43,44].

No.	Functional Category	No. of IRFIs	Representative Ingredients
1	Intestinal health	14	*Bacillus coagulans* SNZ 1969 probiotics, *Bacillus coagulans* Unique IS-2 probiotics, Coffee mannooligosaccharides powder, Fig paste, Galactooligosaccharide powder, Heat-treated *Lacticaseibacillus paracasei* subsp. *paracasei* 327(K-1) culture powder, Isomalto-oligosaccharide, Lactulose powder, PME88 melon extract, *Portulaca oleracea* L. extract powder, Probiotics (De simone), Resistant maltodextrin from wheat starch, Soy oligosaccharide, Xylooligosaccharide powder
2	Blood glucose control	20	Albumin, Black soybean peptide, Chitooligosaccharide, Fermented soy bean extract, Freeze dry silkworm powder, Hydrolyzed ginseng extract, Hydroxypropyl methyl cellulose, *Lactiplantibacillus plantarum* HAC01 probiotics, L-arabinose, Mixture complex of Mori Folium and Aurantii Fructus extract, *Morus alba* L. Branch ethanol extract powder, Mulberry leaf extract, Nopal extract, Pine needle distilled concentrate, Powder of *Capsicum annuum* L. cv. Dangjo, Red ginseng, Seapolynol *Ecklonia cava* ethanol extract, Tagatose, The submerged culture of *Ceriporia lacerata* mycelium, Unripe *Momordica charantia* ethanolic extract powder
3	Joint and bone health	42	*Andrographis paniculata* Wall. ex Nees extract, *Angelica gigas* extract powder (Nutragen), *Anthriscus sylvestris*(L.) Hoffm leaf extract, Bees wax alcohol, Boswellia extract (FJH-BS), *Boswellia serrata* extract, *Boswellia serrata* R. extract, Chicken breast cartilage powder, Chondroitin sulfate sodium, *Chrysanthemum* extract, Complex of krill oil, Hemetococcus extract and sodium hyaluronate, *Curcuma longa* root extract, Egg shell membrane hydrolysate, Extract complex of *Achyranthes*, *Eucommiaceae* and pomegranate, Extract complex of fermented *Achyranthes*, *Angelica gigas* and *Eucommia*, Extract complex of *Schisandra chinensis*, *Eucommia ulmoides* and *Lycium chinense*, Extract complex of *Boswellia serrata*, *Curcuma longa*, and *Terminalia chebula*., Extract complex of *Tamarindus indica* and *Curcuma longa*, Extract powder of *Lilium lancifolium* Thunb., Fatty acid complex, Fatty acid complex containing cetyl myristoleate, Ginseng extract, Hop extract, Hyaluronic acid, Krill oil, *Lactobacillus sakei* LB-P12 probiotics, *Litsea japonica* fruit ethanol extracts, Low-molecular-weight collagen peptide, Milk basic protein, *Panax notoginseng*, Rehmanniae Radix and Acanthopanacis Cortex extract powder, *Perilla frutescens* var. acuta Kudo extract complex, Powder of egg shell membrane, Rehmanniae Radix and Acanthopanacis Cortex extract powder, Rosehip powder, Salmon milt extract, Salmon nasal cartilage extract, *Schisandra chinensis* extract, *Scutellaria* composition, Siberian ginseng mixture extract, *Siraitia grosvenorii* extract powder, Taheebo extract, *Terminalia chebula* Retzins extract
4	Cholesterol improvement	14	*B. breve* IDCC 4401(BBR4401) heat treatment culture powder, Barley β-glucan extract, Bokbunja extract, Changnyeong onion extract, Fermented-red grape concentrate, Flax plant powder, Mixture of *L. plantarum* CECT 7527, CECT 7528 and CECT 7529, *Monascus purpureus*, Plant stanol ester, Policosanol–sugar cane wax alcohol, Puer tea extract, Seapolynol *Ecklonia cava* ethanol extract, Sugarcane wax alcohol, Yellow rice with *Aspergillus terreus*
5	Body fat reduction	56	*Adenophora triphylla* var. *japonica* Hara extract, *Amomum villosum* extract, APIC soybean embryos extract, Artichoke dry aqueous extract, *Aster spathulifolius* extract, *Aster spathulifolius* leaf extract, *Bifidobacterium breve* B-3 probiotic, Black cumin seed extract powder, Black soy peptide, *Cissus quadrangularis* extract, Complex extract of *Undaria pinnatifida*, Diacylglyceride, *Dichrostachys glomerata* extract, Ethanol-extracted powder of *Gynostemma pentaphyllum* leaf, Extract complex of grapefruit, orange and guarana, Extract complex of Lemon verbena and *Hibiscus*, Extract complex of lime and cacao, Extract complex of *Atractylodes macrocephala* Koidzumi and *amomum villosum* Loureiro, Extract complex of *Cornus officinalis* and *Ribes fasciculatum*, Extract of *Gelidium elegans*, Extract of unripe apple, Fermented *Castanea crenata* inner shell extract, Fingerroot extract powder, Fingerroot extract (Panduratin), Green coffee bean extract, Green mate extract, *Irvingia gabonensis* seed extract, *Kaempferia parviflora* extract, Krill oil, *Lactiplantibacillus plantarum* ATG-K2 probiotics, *Lactiplantibacillus plantarum* LMT1-48 probiotics, *Lactobacillus gasseri* BNR17, Lactoferrin, L-carnitine tartrate, *Lonicera caerulea* L. fruits extract powder, Low-molecular-weight whey peptide, *Lythrum salicaria* extract, Marmelo extract, MCFA, Mixture complex of lemonbalm extract powder, Mixture of CJ *Hibiscus* and other extracts, Moroorange extract, Oil containing MCFA, Perilla leaf extract, Probiotics mixture of *L. curvatus* HY7601 and *L. plantarum* KY1032, Puer tea extract, Roasted citrus peel extract powder, Salacia reticulata root extract, *Schisandra chinensis* pomace extract, Skate skin collagen peptide, Sunflower seed extract, The hot water extract of hydrangea leaf, Vinegar-pomegranate complex, Wasabi leaf extract, *Zea mays* and *Melissa officinalis* extract
6	Immune function	15	AHCC, *Bacillus subtilis* chungkookjang culture purification substance, *Cordyceps militaris* extract, Echinacea extract, Enzyme-treated extract of *Cervus elaphus* L., Extract complex of *Perilla frutescens* var. *japonica* (Hassk.) H. Hara and *Portulaca oleracea* L., *Ganoderma lucidum* mushroom mycelia extract, Ginseng polysaccharide extract, *Lacticaseibacillus paracasei* HY7017 probiotics, Lactoferrin, PLAG, Silk protein acid hydrolysate, *Weissella cibaria* JW15 probiotics, Yeast β-glucan, β-glucan powder
7	Antioxidant function	6	Barley shoot extract, Beeswax alcohol, Bokbunja powder, Grape seed extract, PME88 melon extract, Pycnogenol-French maritime pine bark extract
8	Skin health	49	Agastache rugosa extract, AP collagen peptide, Bonito elastin peptide, CJ Low-molecular-weight collagen peptide, Collactive collagen peptide, Concentrated powder of milk cream, Concentrated powder of *Citrus sinensis* (L.) Osbeck (ROCH), Corn germ extract, Dandelion extract powder complex, *Elaeagnus umbellata* Thunberg fruits extract, Extract complex of Indian gooseberry and barley sprout, Extract complex of rosemary and grapefruit, Extract complex of *Rosmarinus officinalis* L. and *Tagetes erecta* L., Extract of *Actinidia arguta*, Fermented barley and soybean moisturizing agent, Fermented honeybush extract powder, Fingerroot extract powder, Fingerroot extract, Fish collagen peptide, Galactooligosaccharides, Gromwell extract, Korean mint, Goji berry and fig mixed extract, Krill Oil, Low-molecular-weight collagen peptide AG, Low-molecular-weight collagen peptide GT, Low-molecular-weight collagen peptide NS, Low-molecular-weight collagen peptide SH, Low-molecular-weight fish collagen peptide, Low-molecular-weight porcine placenta peptide, Low-molecular-weight collagen peptide, Oyster hydrolysate, Pine bark extract complex, PME88 melon extract, Pomegranate juice concentrate powder, Powder of egg shell membrane, Probiotics HY7714, Probiotics mixture of L. plantarum PBS067, *L. reuteri* PBS072 and *L. rhamnosus* PBS070, Red ginseng, *Torilis japonica*, *Cornus officinalis* complex extract, Refined fish oil from Alaska pollak containing palmitoleic acid, Rice bran extract, Rose petal extract, Silver vine fruit extract powder, The hot water extract of hydrangea leaf, Unripe apple concentrated powder, *Vaccinium uliginosum* L. fruits extract powder, Wheat endosperm extract, Wheat extract, Whey protein lipid extract
9	Blood pressure control	13	Black raspberry extract powder, Black soy peptide, *Eleutherococcus sessiliflorus* fruits extract powder, Enzyme treated grape seed extract, Enzyme treated red ginseng extract powder, Fermentation powder of the *Bacillus subtilis* var. *natto*, GABA from L-glutamic acid powder, Hydrolyzed casein, Katsuobushi oligopeptide, Nori peptide, Olive leaf extract, Policosanol–sugar cane wax alcohol, Salmon peptide, Sardine peptide SP100N
10	Triglyceride reduction	3	*Lactiplantibacillus plantarum* Q180, Post-fermented tea (Heukcha) extracts, Refined squid oil
11	Blood circulation improvement	10	Fermentation powder of the *Bacillus subtilis* var. *natto*, HK natto culture extract, L-arginine, Plant extract mixture of *Phellinus baumii* and *Salvia miltiorrhiza* Bunge, PME88 melon extract, Policosanol–sugar cane wax alcohol, Pycnogenol-French maritime pine bark extract, Refined squid oil, *Theobroma cacao* L., *Vitis labrusca* L. leaf extract
12	Memory enhancement	13	*Angelica gigas* Nakai, *Saururus chinensis* Baill, *Schisandra chinensis* extract complex, BF-7 (Brain Factor-7) silk fibroin peptide, *Eriobotrya* leaf extract, Extract powder of *Panax ginseng* C.A. Meyer sprout, Fermented sea tangle, *Gastrodia* combination extract, Heat treated green tea extract, *Lycium chinense* fruit extract, Mixture complex of grape and blueberry powder, *Polygala tenuifolia* Willdenow root extract powder, *Scrophularia buergeriana* Miquel extract, Sesame oil cake extract, Spirulina extract
13	Liver health	14	*Artemisia annua* extract powder, *Centella asiatica* extract, *Ecklonia stolonifera* extract, Extract complex of lemon balm and dandelion, Extract complex of *Hovenia dulcis* and *Schisandra chinensis*, Fermented sea tangle, Ginseng, HK shiitake mushroom mycelia, *Hovenia dulcis* Thunb. extract powder, *Lonicera caerulea* L. fruit (honeyberry) extract powder, *Lonicera caerulea* L. fruits (honeyberries) extract powder, *Mangus Lentinus edodes* mycelia extract powder, *Platycodon* root extract, Probiotics fermented garlic extract powder, Probiotics mixture of *L. plantarum* LC27 and *B. longum* LC67
14	Eye health	15	Bilberry extract, *Centella asiatica* extract, Complex of lutein and zeaxanthin extracted from *Tagetes erecta* L., Dried powder of *Tetraselmis chuii*, Ethanol extract powder of *Diospyros kaki* leaves, Extract of *Perilla frutescens* var. *acuta* Kudo, Grape skin enzyme fermentation extract, Lutein zeaxanthin complex extract, Lutein zeaxanthin complex extract 20%, Maquiberry extract, Marigold extract, Marigold flower ethanol extract (containing zeaxanthin), Marigold flower extract, Small black soybean extract, *Tisochrysis lutea* powder
15	Stress relief	4	*Ashwagandha* extract, *Gynostemma pentaphyllum* Makino extract powder, *Ocimum tenuiflorum* extract, *Saffron* extract
16	Cognitive function improvement	10	*Angelica gigas* extract powder, *Angelica gigas* root extract, *Aster glehni* extract powder, Complex of *Lactiplantibacillus plantarum* C29 probiotics and fermented soybean powder, Enzyme-treated *Tremella fuciformis* extract, *Gastrodia elata* extract, Leaf and stem extract of *Dendropanax morbiferus*, Probiotics mixture of *L. mucosae* NK41 and *B. longum* NK46, Pyrroloquinoline quinone disodium salt, *Turmeric* extract
17	Prostate health	6	Complex extract of *Cervus elaphus*, *Angelica gigas* and *Glycyrrhiza uralensis*, Extracts complex of *Angelica gigas* Nakai and *Astragalus membranaceus* Bunge, *Quisqualis indica* Linn. extract, Red ginseng oil, *Salvia miltiorrhiza* extract, Saw palmetto extract complex
18	Calcium absorption	0	-
19	Exercise performance	4	CaHMB, Fermented of *P. hepiali (Cs-4)* from *Cordyceps sinensis*, *Hovenia dulcis* Thunb. extract powder, *Lactiplantibacillus plantarum* TWK10 probiotics
20	Urinary tract health	2	Extract of cranberry, Pacran cranberry powder
21	Dental health	1	Xylitol
22	Fatigue recovery	5	Complex extract of *Angelica gigas* Nakai, *Cervus elaphus* L. and *Astragalus membranaceus* Bunge, Fermented generating amino acid complex, *Hovenia dulcis* Thunb. extract powder, L-carnitine tartrate, The fermented extract of pig placenta
23	Male menopause support	9	Complex extract of *Eucommia ulmoides* Oliver and *Achyranthes japonica* Nakai (*Achyranthes bidentata* Blume), Extract complex of fenugreek seed and Lespedeza, Fenugreek seed extract, Fermented soy germ powder (SE5-OH), Korean dandelion extract complex, *Lespedeza cuneata* G. Don extract, Maca gelatinized powder, *Rhus verniciflua* extract powder, *Trigonella foenum-graecum* seed extract
24	Female menopause support	11	EstroG-100, Ethanol extract powder of the root of *Salvia miltiorrhiza* Bunge, Extract complex of soybean and hop, Fermented germinated soybean extract, *Lactobacillus acidophilus* YT1, MS-10 thistle complex extract, Pomegranate extract, Pomegranate juice concentrate, Pycnogenol-French maritime pine bark extract, Rhapontic rhubarb root extract, *Schisandra chinensis* extract
25	Sensitive skin improvement	3	Fruit and vegetable origin LAB (*L. plantarum* CJLP133), *L. sakei* probio65, *Lacticaseibacillus rhamnosus* IDCC3201 heat treatment culture powder
26	Urination function	1	Complex of pumpkin seed extract et al.
27	Gastric health	13	Bees wax alcohol, *Cudrania tricuspidata* leaf extract, *Dolichos lablab* L. extract powder, Extract complex of *Rubus crataegifolius*, Extract powder of the leaf of *Artemisia argyi*, Fermented gold kiwi, Honeysuckle flower extract (greencera-F), *Lactobacillus* fermented soybean powder, Licorice extract, Mastic gum, Mixture of extracts from *Paeonia lactiflora* and *Inula britannica*, Salmon milt extract, Stemed *zingiber officinale* Roscoe extract
28	Sperm motility	1	Maca gelatinized powder
29	Vaginal microbiome support	3	Licorice extract, Respecta (probiotic complex), UREX probiotics
30	Premenstrual symptom relief	1	Extract complex of *Hordeum vulgare* and *Chrysanthemum zawadskii*
31	Height growth in children	3	Extract of *Humulus japonicus* Siebold et Zucc., Fermented oyster extract, Mixture of *Astragalus membranaceus*, *Phlomis umbrosa* and *Eleutherococcus senticosus*
32	Sleep quality improvement	8	*Ashwagandha* extract, *Ecklonia cava* ethanol extract, Fermented L-glutamate GABA powder, Fermented rice germ powder, Lettuce extract, Lime peel extract, Milk protein hydrolysate (Lactium), Rice bran ethanol extract
33	Muscle strength improvement	8	*Curcuma longa* extract, Extract powder of the root of *Lithospermum erythrorhizon*, Fermented oyster extract, Fermented velvet antler (*Cervus elaphus* L.) powder, Low-molecular-weight whey peptide, Low-molecular whey protein hydrolysate, Pasteurized *Akkermansia muciniphila* HB05 (HB05P) powder, *Schisandra chinensis* extract
34	Leg discomfort (swelling/edema)	0	-
35	Respiratory tract/bronchial condition (cough, phlegm, etc.)	3	Extract complex of *Chrysanthemum indicum* L. and *Ecklonia cava*, Heat-treated *Akkermansia muciniphila* EB-AMDK19 culture powder, Mixture of probiotics (*Lactiplantibacillus plantarum* KC3) and *Leonurus japonicus* Houttuyn extract (CKDB-315)
36	Uremic toxins	0	-
37	Hearing maintenance	0	-
38	Bad breath (halitosis)	0	-
39	Hair health	4	Complex of extract from *Panicum miliaceum* L. and *Triticum aestivum* L., Fish collagen peptide, *Latilactobacillus curvatus* LB-P9 probiotics, Low-molecular-weight collagen peptide
40	Gum health	1	Extract complex of propolis and *Garcinia mangostana* L.
41	Immune hypersensitivity	8	*Aster yomena* extract powder, *Enterococcus faecalis* extract powder (LFK), Extract of *Actinidia arguta*, Extract of *Saururus chinensis* Baill, Guava leaf complex extract, Immature *Canavalia gladiata* extracts, Mixture of *L. plantarum* IM76 and *B. longum* IM55, Picao preto powder blend

**Table 3:** Comparison of HFF regulatory systems across major countries [12,41,43,61,62,63,64,65,66,67,68,69].

Country	Regulatory System	Pre-Market Review	Evidence Required for Functional Claims	Exclusive Use by Company	Approval Timeline
Republic of Korea	IRFI	Yes	Human intervention trials required	Allowed	≥120 days
United States	NDI	Partial	Primarily safety data	Allowed	≥75 days
European Union	EFSA	Yes (EFSA evaluation)	Human RCT-based substantiation	Not allowed	≥9 months
Japan	FFC	No (self-notification)	Literature review or human trials	Practically possible	≥60 days
China	Health Food Registration System	Yes	Functional efficacy evidence required	Conditionally allowed	12–24 months (registration), 6–12 months (filling)

**Table 4:** List of MFDA-approved IRFIs and globally known supplements with reported or suspected DDSIs.

IRFIs or Globally Known Supplements	Potential Interactions	Mechanisms	Types of Evidence	Refs.
Red ginseng	Warfarin, antiplatelet drugs, antidiabetic agents	CYP2C9 modulation, platelet inhibition, glucose-lowering synergy	Human case reports, clinical interaction studies	[106,107]
*Ginkgo biloba* Extract	Warfarin, antiplatelet drugs, SSRIs, anticonvulsants	Platelet-activating factor inhibition; CYP2C19 and CYP3A4 modulation	Multiple case reports, clinical studies, meta-analyses	[97,98]
Milk Thistle (*Silybum marianum;* Silymarin)	Statins, antidiabetic drugs, immunosuppressants	CYP3A4, CYP2C9, UGT modulation; P-gp effects	In vitro studies, pharmacokinetic trials	[93,100]
Natto fermentation products	Anticoagulants (warfarin), antiplatelet drugs (aspirin, clopidogrel)	Fibrinolytic activity; clotting factor modulation	In clinical studies, enhanced fibrinolysis	[108,109]
Red Yeast Rice (Monacolin K)	Statins, CYP3A4 substrates, antifungals	Same metabolic pathway as lovastatin	Clinical DDSIs analyses, case reports	[110,111]
L-Theanine (Green Tea Extract)	Antihypertensives, stimulants, caffeine-containing drugs	Blood pressure modulation, CNS activity changes	Human studies and pharmacodynamic interactions	[105,106]
GABA (Gamma-Aminobutyric Acid)	Sedatives, benzodiazepines, antihypertensive drugs	CNS depressant synergy, blood pressure lowering	Efficacy	[112,113]
Ashwagandha (*Withania somnifera*)	Sedatives, thyroid medications, immunosuppressants	GABAergic modulation, thyroid hormone changes	Clinical trials and case reports	[114,115]
Curcumin (*Curcuma longa* root extract)	Anticoagulants, antiplatelets, CYP3A4/CYP1A2 substrates	CYP inhibition, anti-inflammatory synergy	interaction studies, case reports	[116,117]
Probiotics (e.g., *Lactobacillus* TWK10, *Weissella* JW15)	Antibiotics	Reduced probiotic viability; altered absorption	Known DDSIs mechanism, although mild	[118,119]
Cordyceps Extract	Immunosuppressants, anticoagulants, antidiabetic drugs	Immunomodulatory and glucose-lowering effects	PD/PK interaction concerns reported	[120,121]
Pomegranate Extract	CYP3A4 substrates (similar to grapefruit in some studies)	CYP3A inhibition (controversial but noted)	Conflicting evidence; included due to potential mechanistic overlap	[122,123]
Marigold Extract (Lutein/Zeaxanthin)	Fat-soluble drug absorption competition	Shared lipid transport pathways	Clinical absorption studies	[124,125]

**Table 5:** Comparison of regulatory guidelines for DDSIs assessment of HFFs (FDA, EFSA, MFDS) [23,62,78].

Regulatory Body	Pre-Market Interaction Assessment	Mandatory DDSIs Data	Evidence Emphasis	Regulatory Strength	Key Features
FDA (U.S.)	Not required for supplements; required for NDI	No (safety-focused only)	CYP/Transporter interaction, safety data	Moderate	Strong labeling rules; adverse event reporting
EFSA (EU)	Required for Novel Food or Health Claim applications	Yes, when relevant	Human RCTs, mechanistic plausibility	Strongest scientific rigor	No exclusivity; EFSA scientific substantiation
MFDS (Korea)	Integrated within safety evaluation; not standalone	Not mandatory	ADME review, clinical study design considerations	Medium–High	Unique exclusive-use system; post-market re-evaluation

## Data Availability

No new data were created or analyzed in this study. Data sharing is not applicable to this article.
